# Burnout and Nursing Care: A Concept Paper

**DOI:** 10.3390/nursrep12030044

**Published:** 2022-07-03

**Authors:** Vitor Parola, Adriana Coelho, Hugo Neves, Rafael A. Bernardes, Joana Pereira Sousa, Nuno Catela

**Affiliations:** 1 The Health Sciences Research Unit: Nursing (UICISA:E), Nursing School of Coimbra (ESEnfC), 3004-011 Coimbra, Portugal; adriananevescoelho@esenfc.pt (A.C.); hugoneves@esenfc.pt (H.N.); rafaelalvesbernardes@esenfc.pt (R.A.B.); 2 Center for Innovative Care and Health Technology—ciTechCare, School of Health Sciences, Polytechnic of Leiria, 2411-901 Leiria, Portugal; joana.sousa@ipleiria.pt; 3 School of Health Sciences, Polytechnic of Leiria, 2411-901 Leiria, Portugal; nuno.correia@ipleiria.pt

**Keywords:** nursing, nurse–patient relations, burnout

## Abstract

Burnout comprises a series of undetermined physical and psychosocial symptoms caused by an excessive energy requirement at work—it is a crisis in relationships with work itself and not necessarily a concern with underlying clinical disorders related to workers. Professions involving human interactions commonly involve emotional engagement, especially when the cared-for person needs assistance and support, as is the primary concern in the nursing profession. To some extent, the acknowledgment of the phenomena of burnout and how it affects people is sometimes addressed from a biomedical perspective. This concept paper aims to describe the burnout concept and reflect on the impact on nurses. Our intention with this reflection, considering the burnout impact on nurses, is to support a paradigm change in the prevention and management of burnout in healthcare contexts, promoting and fostering the well-being of nurses.

## 1. Introduction

The burnout concept was first mentioned by Herbert Freudenberg [1], in the 1970s, as involving a series of unspecific physical and psychosocial symptoms generated by an excessive energy requirement at work. This first definition of the concept served to identify, describe, and name an existing social problem based on observations. Nevertheless, those observations were not systematic or standardized [2]. However, with the development of standardized instruments in the 1980s, such as the Maslach Burnout Inventory and Burnout Measure, burnout started to be studied empirically.

Maslach and Leiter [3] extended the concept of burnout and redefined it as a crisis in interactions with work and not necessarily a concern with working people. Burnout is believed to result from continued exposure to work-related stressful events [4]. Maslach and colleagues indicated that burnout research is rooted in caregiving and service occupations, where the central core of the job is the relationship between the cared person and the caregiver [2,5,6]. Professions involving human interactions commonly involve emotional engagement, especially when the cared-for person needs assistance and support, as is the primary concern in the nursing profession.

According to Maslach and Leiter [3,5], burnout is characterized as a syndrome with three dimensions: ‘emotional exhaustion’, ‘depersonalization’, and a ‘lack of personal accomplishment at the workplace’ that occur when functional coping strategies fail. These dimensions are further explained.

Regarding ‘emotional exhaustion’, it arises when health professionals reach the limits of their capacity. As a result, there is a lack of emotional energy and a perception that emotional resources are depleting. For that reason, professionals cannot respond at an emotional level [2,5]. ‘Emotional exhaustion’ is the reaction to chronic stressors in the workplace, such as work overload, which are constant over time and introduce a pressure component on people’s daily lives, causing emotional exhaustion. It is the lack of emotional energy, not directly physical energy itself [2,5,7]. People are not physically fatigued from performing a strenuous job; the main issue is being emotionally drained from the lack of resources to deal with demands and stressors. The exhaustion increases the possibility of distancing oneself emotionally and cognitively from work, apparently as a way to cope with work overload. This lack of energy, perceived as a further loss of resources, can lead to maladaptive coping strategies such as emotional detachment from work or depersonalization [2,4,5].

Regarding ‘depersonalization’, it is defined as an impersonal and distant contact, where the nurse, for example, starts to use remote approaches toward patients and colleagues, actively ignoring the other’s unique and engaging qualities and developing negative feelings and cynical attitudes—a reason why the term ‘depersonalization’ is often synonymous with cynicism in the burnout literature. Depersonalization usually develops due to increased exhaustion, being self-protective at the beginning—an emotional defense of ‘detached concern’. It is seen as a coping mechanism because it distances workers from the job and other people, such as colleagues and/or patients. In the case of health care professionals, who evidence depersonalization attitudes at their job, the human service workers attempt to block negative emotions, decreasing emotional exhaustion and recovering resources, which increases energy [4,5,8,9].

Distancing arises as a coping mechanism to emotional exhaustion, disengaging the person from work and preventing additional emotional exhaustion. An attempt to cope with emotional exhaustion by becoming emotionally detached using distancing occurs. However, the consequence is that the detachment is capable of causing the loss of idealism and the dehumanization of others. With time, the nurse is not only creating a shield and cutting back on the amount of work but also creating an adverse response to others and to professional tasks and responsibilities. As a result, the nurse shifts from trying to do his/her very best to doing the bare minimum [2,3,4,5,10].

The ‘lack of personal accomplishment’ usually refers to negative feelings about competence and professional success, evidencing a lack of motivation and decreased productivity at work [2,5]. This dimension represents the self-evaluation component of burnout. For example, an expectable part of a nurse’s job is to care for others. Still, if the nurse is emotionally exhausted and depersonalizing his/her surrounding, he/she will perceive the tasks as inadequate, lacking in personal accomplishment, and reducing one’s perceived professional efficacy [2,3,4,5]. This sense of inefficacy may lead nurses affected by burnout to a severe dislike of the kind of person they think they have become, leading to a loss of confidence and an increased risk of having negative self-esteem [2,4,5].

At this point it is important to note that the most widely used concept of burnout (Maslach and Leiter) refers to these three dimensions. However, in the use of the instrument developed by the authors, it is often mentioned that burnout is considered to be present if (a) the three subscales are altered; (b) emotional exhaustion and/or the depersonalization scale are altered (the sub-scale personal accomplishment not being taken into consideration and a high score for one of the other two sub-scales are enough to be considered as burnout); and (c) there is at least one dimension with severely abnormal ratings [11].

Worldwide there are several studies about the incidence and prevalence of burnout. A survey carried out with intensive care nurses reveals that nurses reported a high level of emotional exhaustion (73.9%) and depersonalization (52.2%), and a medium level of personal accomplishment (40%) [12]. Another study evidenced a high burnout prevalence (70%) among nurses during the peak of the first wave of the COVID-19 pandemic [13]. Moreover, evidence shows that nearly half, 49.1% (*n* = 194), of hospital bases nurses had high levels of burnout [14].

In summary, we can say that burnout is composed of three dimensions: emotional exhaustion, depersonalization, and a lack of personal accomplishment, which are related, with the lack of personal accomplishment dimension being more related to a nurse’s self-evaluation.

## 2. Materials and Methods

A conceptual, descriptive study was carried out to analyze and interpret sources published about the concept of burnout. According to a structural perspective, contextualization of the analysis produced regarding burnout in the nursing profession was complemented with the necessary contextual analysis of health contexts. An interpretative text was formulated with the necessary conclusions.

Studies were selected on the basis of a search conducted in Medline (via Pubmed), CINAHL with full text (via EBSCOHost), and Scopus, using keywords and Mesh-terms/CINAHL-headings adjusted to the respective databases. Two types of articles were chosen for this paper: reference documents and articles in the field to describe the concept; articles had to be published in recent years (5 years, prioritizing the last year) for specific aspects, for example, interventions/programs discussed or the impact of burnout, as they are more adjusted to the current reality and evidence current trends in the topic. The search strategy used for Medline (via PubMed) is presented in Table 1. 

## 3. Results and Discussion

### From Burnout Conceptualization to Maslach and Leiter’s Areas of Work-Life Model

Considering the three dimensions previously described, Maslach and Leiter [4] mentioned that the experience and etiology of burnout build based on exhaustion levels, starting a landslide and resulting in a personal career crisis.

Cynicism and depersonalization take the experience of exhaustion to a higher level and are compounded by inefficacy. Instead of giving the possible satisfaction, fulfilment, and validation of one’s identity, working in the healthcare sector becomes a joyless burden in need of minimization, avoidance, and escape. This could explain why burnout was significantly associated with depression in nurses [15]. 

In this sense, when a workplace is noticed as exceptionally demanding, emotional, mental, and spiritual exhaustion can occur because of a concomitant decrease in the levels of energy and confidence [4,10]. Eventually, workers’ enthusiasm, organizational commitment, and dedication to their work vanish, influencing nurses’ performance, quality of life, job satisfaction, and global personal health [2,16]. A recent systematic review about unfinished nursing care shows that working in highly demanding environments is associated with reduced job satisfaction, burnout, and intention to leave [17]. Given the challenges in nurse satisfaction, recruitment, and retention, future research needs to focus on nurses’ quality of work.

Some aspects should be clarified when discussing burnout since they are interrelated concepts. One of these aspects is the distinction between burnout and stress. The difference between them is a question of time. Burnout refers to the long-term breakdown in adaptation complemented by chronic malfunctioning at the workplace. A nurse who suffered from job stress would return to normal; however, one suffering from burnout would not do so since burnout results from chronic stressors in the workplace [6,7].

Another aspect to clarify is the possible misunderstanding between burnout and compassion fatigue concepts. The latter is frequently thought of as the caregiver’s cost of caring but occurs when nurses are exposed to recurrent interactions that demand high empathic commitment with distressed patients. At present, compassion fatigue can be a substantial influencing factor in nursing burnout [18]; several studies detected a significant positive correlation between compassion fatigue and burnout [19,20]. However, there is no systematic review on this topic. For example, education, awareness, and self-care are key elements in preventing compassion fatigue [18].

Having clarified the distinction between burnout, stress, and compassion fatigue, it is relevant to mention that other themes are also related to burnout (e.g., depression, anxiety, workload, work performance, co-worker relationships, quality of life), and they should be considered [19,20,21]

As stated earlier in this paper, nursing is a stressful profession dealing with human health and illness, eventually leading to job dissatisfaction and burnout [22,23]. When they care for people, the impact on nurses should be acknowledged. For that reason, nurses should recognize early signs of burnout and seek appropriate help [24]. The strategies for combating burnout are related to changing healthcare systems to provide support for nurses. To prevent this, institutions may explore alternative work schedules and lower patient loads [18].

The experience of burnout has been related to an extensive list of adverse outcomes, namely at a personal, social, and organizational level. These outcomes involve more medical errors and poor quality of patient care in healthcare [4]. Therefore, it is not unreasonable to presume that nurses’ burnout interferes with their performance and then with the care process [4,25,26]. Maslach and Leiter [4] state that when hospital staff experience high levels of burnout, their patients present lower satisfaction levels with the received care. The literature reveals a strong correlation between low personal accomplishment scores and the poor care behaviors of nurses [12].

A cross-sectional survey among nurses showed a positive correlation between emotional exhaustion, depersonalization scores, and patient care quality [27]. Another cross-sectional study in long-term care wards established an association between nurses’ burnout and objective care quality indicators. Higher emotional exhaustion was associated with statistically significant higher rates of pneumonia and pressure ulcers, and reduced personal accomplishment was associated with higher tube feeding rates [28]. In recent systematics reviews and meta-analyses about the influence of burnout on patient safety, the authors have revealed a relationship between high levels of burnout and worsening patient safety [29,30].

Burnout is additionally related to dysfunctional relationships with co-workers and a deeper intention to leave the health profession altogether [4,17]. For that reason, Maslach and Leiter [4], in their reflection, argue that it is urgent to address burnout levels among professionals, not only because of the natural discomfort of such an event but also because of other severe consequences at the workplace.

The majority of the research evidence indicates that burnout does not suggest something is wrong with the professional, but rather that there has been a fundamental shift in the workplace and the nature of the job. It follows that burnout does not begin as a personal failure [3,4,7], but develops in response to challenging relations among employees and their workplaces, also being a social and organizational issue. Both the person/professional and the organization to which they belong have a role in improving the workplace and people’s performance [3,4,6]. As Montgomery [18] highlighted in his study, burnout is an essential indicator of how the organization functions.

From Maslach and Leiter’s perspective [3], burnout occurs from chronic mismatches between the person and the job in terms of some or all the six areas of work-life (AW), described in Table 2.

The AW model indicates management areas in which professionals encounter disappointments that increase burnout levels. As Maslach and Leiter [7] mention, some mismatches in the areas described above impact an individual’s level of experienced burnout, which, in turn, determines numerous outcomes, such as job performance, social behaviors, and personal health. In a sense, the greater the disparity between person and profession, the greater the likelihood of developing a degree of burnout. One of the advantages of the AW model is that any of the six areas can be improved [4,7,31]. Additionally, some processes enhance the alignment of people with their work settings, holding great potential for positive changes [4,17].

Addressing burnout includes interventions to alleviate it when events arise and also to prevent it before an event occurs [4,7,24]. The first type of intervention occurs with individuals or workgroups are feeling levels of burnout that, even when high, are not necessarily serious enough to prevent them from working. Conversely, prevention strategies tend to concentrate on professionals generally not affected by burnout, helping them avoid the risk of developing burnout events [4,32].

Some individual strategies were adapted from previous studies on stress, coping, and health topics. Notably, (i) altering work patterns (e.g., working fewer hours, avoiding overtime work, balancing work with personal life); (ii) improving coping skills (e.g., cognitive restructuring, conflict resolution, time management); (iii) getting social support (from colleagues and family); (iv) using relaxation strategies; (v) encouraging good health and fitness; and (vi) developing a better self-understanding through diverse self-analytic techniques, counselling, or therapy [7,33]. As a practical approach, we could pose that if an intervention is more focused on the sense of efficacy, it might better respond to improvements, such as a more clear culture of recognition from colleagues and leaders [7].

Some studies have made further recommendations to cope with burnout. For example, the need to care for oneself, and not just about individual health and physical fitness, but also about psychological well-being. Other recommendations include encouraging a focus on spirituality and human nature; encouraging better social recognition of the challenging work being accomplished; and concentrating on the good aspects of life, at work and at home [7,22,34].

Considering the impact of the subject under analysis, which has been demonstrated in several studies, one of the highlighted aspects is the extreme physical and emotional demands requested from nurses. It is not surprising that there has been a growing number of studies on this topic about the magnitude of burnout during the pandemic because of the increased demand from all professionals [35,36,37]. That is why it is necessary to know this reality and create mechanisms that minimize its impact so that we are prepared as much as possible to respond to it, especially in situations where its incidence increases considerably [38].

## 4. Conclusions

When nurses focus on patients, they may forget to care for themselves. In this sense, this concept paper evidences the need for emotional support for healthcare team members. Furthermore, it is essential that evidence clearly explains the strategies nurses adopt, or should adopt, to help them in daily caring interventions. In addition, a clear understanding of the factors that affect nurses will directly impact the levels of emotional exhaustion, depersonalization, and personal accomplishment, which generate burnout. Thus, a deeper understanding of how nurses manage these factors and which strategies nurses adopt or should adopt is required.

As stressed by Maslach and colleagues, burnout research had its origins in professions involving human interactions, especially when the cared-for person needs assistance and support, as is the main concern in the nursing profession [3,4,7,39]. This article discussed the existing evidence to support a paradigm change in the prevention and management of burnout in healthcare contexts and provided incentives for future research promoting the well-being of nurses. As was mentioned by the authors, once the levels of burnout are known, it is vital to move towards interventions that mitigate it [8,15,17,18,39]. Institutions and stakeholders should consider this reality to develop appropriate psychological interventions and strategies to prevent, alleviate, or treat burnout among nurses.

Burnout in healthcare services is undoubtedly a critical issue. It needs to be addressed within the workplace, enhancing the teamwork perspective, for example, and the educational process, which prepares people for a healthier career. Therefore, this topic should be on the agenda, as it is essential that nurses and stakeholders know about it, discuss it regularly, and work with others to deal with it.

## Figures and Tables

**Table 1:** Search conducted in Medline (PubMed).

Search (Query)	Record Retrieved
(nurs*[Title/Abstract]) AND ((((((professional burnout[MeSH Terms] OR burnout[Title]) OR (emotional exhaustion[Title])) OR (depersonalization[Title])) OR (personal accomplishment[Title])) OR (cynicism[Title]))) AND ((y_5[Filter]) AND (medline[Filter]) AND (english[Filter] OR portuguese[Filter] OR spanish[Filter]))	1502

**Table 2:** Description of the AW model.

Area	Description
**Workload**	Struggling with more requirements than one person can manage aggravates burnout, particularly the exhaustion dimension of this syndrome. Professionals with a profound commitment to their work suffer significant disappointment when not completing tasks they feel are essential. The tendency of work to spread to their private life generates a distinct burden, in part due to such a situation interrupting opportunities to recover the dwindling energy.
**Control**	It is associated with taking part in decisions affecting a person’s work. People differ in how they seek to exercise choice and control at the workplace. Some people take comfort from others looking after the details, while others feel compelled to make an active contribution to workplace decision-making. Control enables people to exert initiative in their jobs, offering them a sense of agency and volition.
**Reward**	Acknowledgement for one’s contributions in the workplace sets the third AW. Once more, people differ, with a few content with the inherent rewards of their job activity, while others are primarily worried about receiving validation from colleagues and leaders. The degree to which the workplace is aligned with the size and the kind of acknowledgement one pursues impacts one’s susceptibility to burnout.
**Community**	People’s quality relations at work have a central role. People differ in the level that they appreciate close relationships or restricted professional associations within the workplace. Nevertheless, people always prefer favorable social exchanges within whichever mode they want to encounter one another. They may also leave a team when relations are tense between colleagues.
**Fairness**	A feeling of fairness involves individuals with their workplace, while the sense of unfairness depletes and discourages them, urging them to alienate emotionally and physically from work. Unfair treatment excludes individuals from being considered effective members of their workplace communities. Nurses frequently feel indifferent to jobs that they believe are caring for patients unfairly.
**Values**	An alignment of personal and institutional values characterizes the sixth AW. The nature of healthcare as value-focused work makes this particular area relevant to all health workers. Collaboration with a team that shares fundamental values empowers individuals while doing a job that appears futile or even harmful to patients produces exhaustion and depersonalization.

Adapted from Maslach and Leiter [4].

## Data Availability

Not applicable.
